# Subgroup identification for treatment selection in biomarker adaptive design

**DOI:** 10.1186/s12874-015-0098-7

**Published:** 2015-12-09

**Authors:** Tzu-Pin Lu, James J. Chen

**Affiliations:** Division of Bioinformatics and Biostatistics, National Center for Toxicological Research, Food and Drug Administration, 3900 NCTR Road, HFT-20, Jefferson, AR 72079 USA; Department of Public Health, Institute of Epidemiology and Preventive Medicine, National Taiwan University, Taipei, Taiwan; Graduate Institute of Biostatistics, China Medical University, Taichung, Taiwan

**Keywords:** Adaptive signature design, Classification, Personalized medicine, Predictive classifier, Subgroup analysis, Subgroup selection

## Abstract

**Background:**

Advances in molecular technology have shifted new drug development toward targeted therapy for treatments expected to benefit subpopulations of patients. Adaptive signature design (ASD) has been proposed to identify the most suitable target patient subgroup to enhance efficacy of treatment effect. There are two essential aspects in the development of biomarker adaptive designs: 1) an accurate classifier to identify the most appropriate treatment for patients, and 2) statistical tests to detect treatment effect in the relevant population and subpopulations. We propose utilization of classification methods to identity patient subgroups and present a statistical testing strategy to detect treatment effects.

**Methods:**

The diagonal linear discriminant analysis (DLDA) is used to identify targeted and non-targeted subgroups. For binary endpoints, DLDA is directly applied to classify patient into two subgroups; for continuous endpoints, a two-step procedure involving model fitting and determination of a cutoff-point is used for subgroup classification. The proposed strategy includes tests for treatment effect in all patients and in a marker-positive subgroup, with a possible follow-up estimation of treatment effect in the marker-negative subgroup. The proposed method is compared to the ASD classification method using simulated datasets and two publically available cancer datasets.

**Results:**

The DLDA-based classifier performs well in terms of sensitivity, specificity, positive and negative predictive values, and accuracy in the simulation data and the two cancer datasets, with superior accuracy compared to the ASD method. The subgroup testing strategy is shown to be useful in detecting treatment effect in terms of power and control of study-wise error.

**Conclusion:**

Accuracy of a classifier is essential for adaptive designs. A poor classifier not only assigns patients to inappropriate treatments, but also reduces the power of the test, resulting in incorrect conclusions. The proposed procedure provides an effective approach for subgroup identification and subgroup analysis.

## Background

Development of the right drugs for the right patients has been the central goal of personalized medicine. Advancement of molecular technologies provides powerful tools to identify appropriate subpopulations of patients able to benefit from particular treatments. This notion is particularly important for cancer treatments that are currently being developed as targeted therapies [1–3] expected to benefit only a subpopulation of patients. Recently, the FDA addressed “the development of therapeutic products that *depend on* the use of a diagnostic test to meet their labeled safety and effectiveness claims” as “In Vitro (IVD) Companion Diagnostic Devices” [4–6]. A Breakthrough Therapy [7] has also been discussed allowing targeted therapies with promising treatment effects to be prescribed to specific patient subpopulations.

Considerable research has been conducted to develop predictive biomarkers for treatment selection [8–10]. Predictive biomarkers define subpopulations of a patient population as biomarker-positive (g^+^) and biomarker-negative (g^−^) based on genomic profiles and/or disease characteristics. It is generally assumed that the g^+^ patients are good candidates for a particular treatment and g^−^patients are poor candidates. The predictive biomarkers are identified and incorporated into randomized Phase III (or II) trial designs, such as enrichment designs and biomarker-stratified designs. Ideally, these biomarkers have been well studied and various performance characteristics well established before the start of a Phase III trial. However, biomarkers that have been completely validated during Phase II for use in Phase III trials are often unavailable [11, 12]. Clinical trials for targeted drugs can be designed for drug-diagnostic co-development by combining a “trial” for treatment effect with a diagnostic “trial” for patient identification [4, 5, 13]. Furthermore, biomarker adaptive designs can be applied in one clinical study to identify the most suitable target subgroup, based on clinical observations or known biomarkers, and to evaluate the effectiveness of the treatment in the patient subgroup [11–15].

Freidlin et al. [11] proposed a cross-validated “adaptive signature design” (ASD), which consisted of two components: 1) development of a binary classifier to select the g^+^ and g^−^ subgroups for treatment optimization, and 2) a subgroup analysis of treatment effect in the selected g^+^ patients likely to respond to the treatment. This approach provided a substantial improvement by increasing the power of detecting a treatment effect in the targeted patients [11, 14]. The ASD approach offers a novel empirical strategy that efficiently establishes treatment effect in the enriched g^+^ patients. Consequently, the method has been discussed extensively for use in Phase III clinical trials [8, 11–17].

The classifier developed to select the target subgroup has a crucial role in the effectiveness of an adaptive design. A classifier identifys enriched patients for efficacy assessment in the current study and also selects patients for treatment assignment in future studies. In the current study, patients are either assigned to the control or the treatment arm. A classifier with less than 100 % accuracy rate results in misclassification, either false positive and/or false negative errors. False positive error inappropriately assigns the g^−^ patients to an ineffective treatment. False negative error assigns some g^+^ patients to the control arm, excluding them from an effective treatment. Furthermore, in the subsequent subgroup analysis, the estimated differences between treatment and control arms (and between the selected g^+^ and g^−^ subgroups) will be attenuated and the conclusion could be incorrect. A classifier that can accurately assign patients to correct treatment subgroups is the most important consideration of an adaptive design. A clear understanding of the classifier characteristics and the impact of misclassification are essential. However, the issue of classifier performance has seldom been addressed in the development of biomarker-based designs.

The ASD approach was proposed as a supplementary test when the test for all patients is not significant [14]. When the all patient hypothesis is significant, no further tests will be performed. Table 1 of Freidlin et al. [14] showed empirical power when the treatment response rates were 70 % for the g^+^ patients and 25 % for the g^−^ patients. The empirical power for the all patient test was 0.955 if the patient population consisted of 40 % g^+^ patients. That is, 60 % of the patients would receive an unnecessary treatment. Several important issues regarding studies of patients in the g^−^ subgroup (non-responders) are discussed extensively in the FDA enrichment guidance [18]. A test for g^−^ patients will provide useful information for assessment of treatment effects in all patients. Furthermore, the ASD method of Freidlin et al. [11, 14] required two pre-specified tuning parameters to clasify patients into subgroups. The performance of the method depends on the choice of these tuning parameters (details are described below).

This article presents an adaptive design based on the framework of the ASD approach such that the identification of biomarkers and selection of the g^+^ subgroup are planned prospectively in one study. We propose utilizing the diagonal linear discriminant analysis (DLDA) algorithm to classify patients into g^+^ and g^−^ subgroups (Methods). We focus on the performance assessment of classifiers with two aspects: 1) “accuracy” of a classifier to select g^+^ and g^−^ patients, and 2) statistical tests to detect treatment effect in the patient population and subpopulations. The methods are evaluated using simulated data and compared with the ASD method; two public lung cancer datasets are used for illustrative analyses.

## Methods

Consider a randomized clinical trial to compare an untreated control arm with a new treatment arm. To simplify the presentation, we use the term “genomic variables”, including gene expression variables and clinical phenotypic variables. It is assumed that the genomic data are collected before the treatment. Therefore, the treatment should not have effects on these genomic variables. Assume that the sampled patients consist of two subgroups: g^+^ and g^−^. The g^+^ subgroup represents those patients who can benefit from the study treatment. Let π be the proportion of g^+^ patients in the sampled population and (1-π) be the proportion of g^−^ patients, where 0 ≤ π ≤ 1. The primary goal is to identify the g^+^ patients for whom the new treatment is effective and the g^−^ of patients for whom the new treatment is relatively ineffective. Let u_*it*_ denote the response probability for the *i*-th subgroup (*i* = 0 for g^−^ and *i* = 1 for g^+^) in the *t*-th treatment (*t* = 0 for control and *t* = 1 for treatment), where 0 ≤ u_00,_ u_01_, u_10_ ≤ u_11_ ≤ 1.

For a given patient, let z_*k*_ denote the measurement for the *k*-th genomic variable (*k* = 1,…, *m*), and *y*_*kt*_ denote the observed outcome from the predictor z_*k*_ and treatment *t*. The gene expression values z_*k*_ were assumed to be normally distributed with different mean values for predictive biomarker probes between two subgroups and/or between two arms. The response can be binary outcomes, such as “response” or “not-response”, or continuous outcomes, such as disease-free survival time. Following the approach given in Freidlin and Simon [11], the (potential) predictive biomarkers can be identified by fitting a generalized linear regression model [19]:

1 $$ \mathrm{h}\left({\mathrm{y}}_{kt}\right)={\upbeta}_{0k}+{\upbeta}_{2k}t+{\upbeta}_{3k}\left({z}_{k*}t\right)+{\varepsilon}_{kt} $$

where h (y) is a link function. For example, h (.) is the logit link for binary endpoints, identity link for continuous endpoints, and the Cox proportional hazards function [20] for survival endpoints. The interaction coefficient β_*3k*_ measures differential treatment effects for the g^+^ patients compared to the g^−^ patients; a significant interaction β_*3k*_ implies differential treatment responses for the predictor z_*k*_. Let U denote the set of significant genomic variables, denoted as x’s, where the estimated interaction coefficients β_*3k*_’s were significant at a predetermined level. The set U is composed of the true predictive biomarkers showing different expression values between g^+^ and g^−^patients and false-positive probes due to random variation. The set U is used to develop a binary classifier to distinguish g^+^ and g^−^ patients based on the analysis of the response y and significant variables x’s in U.

In the case of a binary response y, the distinction between the two subgroups is self-evident. Numerous classification algorithms have been proposed for subgroup classification [21–25]. We use the DLDA algorithm [24] to classify patients into g^+^ and g^−^ subgroups. The DLDA algorithm has been shown to perform well for high-dimensional data [26], and is robust against imbalanced data [27, 28], a common problem encountered in subgroup classification where the numbers of patients in the g^+^ and g^−^ subgroups differ substantially. Other classifiers, such as random forests [21] and support vector machine [22, 23], may not perform as well if the number of g^+^patients is much smaller than the number of g^−^ patients. The DLDA algorithm is briefly described below.

DLDA is a variant of Fisher’s linear discriminant analysis (LDA) and quadratic discriminant analysis (QDA) [29] which separate samples of distinct groups by maximizing their between-class separability while minimizing their within-class variability. Both LDA and QDA require matric inversion; neither method is directly applicable when the number of predictors is larger than the number of samples. DLDA uses a simple maximum likelihood discriminant rule for a diagonal class covariance matrix with a linear discriminant function; thus, it does not involve matrix inversion. DLDA is robust against imbalanced class-size because the decision boundary for DLDA is based on the sample means and variances of the two classes which are independent of the ratio of class sizes. An R package, sda, is employed for DLDA with the default options.

For continuous response variables, the distinction between the two subgroups is more challenging. Subgroup selection procedures can generally be divided into two steps. The first step is to develop a model to establish the relationship between the response variable y and the predictive variables x’s in U. Specifically, the first step is to develop a model to convert the multiple predictive variables x_*1*_,…,x_*L*_ in U to a univariate predictive score to order each patient’s response, where *L* is the total number of x’s in U. The second step is to find a cutoff-point for the predictive scores to divide the patients into two subgroups.

For survival or disease-free survival, the first step is to fit a Cox proportional hazards model using all variables x’s in U as predictors when the number *L* in U is not large. The estimated regression coefficients b_*l*_’s of the fitted model are the weights of the biomarker variables x_*l*_’s, *l*(**x**) = Σ b_*l*_ x_*l*_. Alternatively, the b_*l*_’s can be estimated using the principal components or standardized test statistics of the variables x_*l*_’s [30]. In the second step, various methods have been proposed for determining the threshold cutoff-point to divide the predictive scores into g^+^ and g^−^ subgroups, such as percentiles of the predictive scores or predictive outcomes [31]. In the analysis of the GSE14814 dataset, estimated survival probability of at least 5 years is considered the univariate predictive score, and probability of 0.5 is used as the cutoff (Results). The DLDA algorithm is subsequently applied to evaluate predictive performance.

For comparison purposes, ASD analysis was conducted using the same data. The ASD method computes the odds ratio of the predicted treatment over control for each predictor variable x_*i*_ in U; for binary responses, the odds ratio is exp{β_*2i*_ + β_*3i*_ x_*i*_}. The ASD method uses a machine learning voting method based on two pre-specified tuning parameters R and G. Specifically, a patient is classified as g^+^ subgroup if the odds ratio exceeds a specified threshold R for at least G predictors in U, that is, exp{β_*2i*_ + β_*3i*_ x_*i*_} > R or β_*2i*_ + β_*3i*_ x_*i*_ > ln(R), x_*i*_ є U.

An important consideration in the development of a class prediction model is evaluation of its performance. The cross validation approach is commonly used to evaluate a classifier’s performance. Typically, the data are divided into training and test sets. The training set is used to select predictive biomarkers and develop the prediction model, and the test set is used to evaluate its performance, including sensitivity, specificity, positive and negative predictive values, and accuracy, in the selection of the g^+^ and g^−^ subgroups. Details of the evaluation of predictive classifiers were given in Baek et al. [26].

Once the g^+^ and g^−^ subgroups are identified, the following comparisons may be carried out: 1) control versus treatment in all patients, 2) control versus treatment in the g^+^ patients, 3) control versus treatment in the g^−^ patients, 4) g^+^ patients versus g^−^ patients within the treatment arm, and 5) g^+^ patients versus g^−^ patients within the control arm. Note that the ASD test strategy [11] started with Comparison 1, and Comparison 2 was only conducted if Comparison 1 was not significant. This can result in unnecessary treatment for g^−^ patients.

The two primary hypotheses are for treatment effect in all patients, Comparisons 1, and in the g^+^ subgroup, Comparison 2. Let α be the pre-specified study-wise error rate. To account for multiple comparisons, the significance levels are set at α_1_ for the overall effect and α_2_ = (α − α_1_) for the subgroup effect. Statistical significance of Comparisons 1 and 2 can conclude that treatment is effective in all patients and in the g^+^ subgroup, respectively. Statistical significance of the treatment effect in the g^−^ subgroup can be directly assessed using Comparison 3. However, in tailoring clinical trials, Comparison 3 is generally statistically insignificant.

Clinical validation/qualification of a biomarker signature and classifier requires prospectively randomized clinical trials to demonstrate treatment efficacy in the classifier identified targeted patients. Thus, Comparisons 2 and 3 are relevant. Comparison 4 addresses the clinical question “Does treatment effect differ between the g^+^ and g^−^ subgroups?” However, this analysis is not a randomized study; also, the observed difference between the two subgroups may be associated with a baseline difference in the control arm, Comparison 5. In many clinical retrospective oncology studies, patients’ allocations to treatments are not random; in these cases, Comparisons 1–5 may not be useful or directly interpretable. However, they are useful for exploratory analysis of treatment effects in the subgroups and between subgroup effects.

In general, candidate predictive biomarkers are often identified initially by multiple retrospective analyses of clinical trials, and a predictive signature is subsequently developed and evaluated in Phase II trials to be used in Phase III trials. Comparisons 4 and 5 are useful for retrospective analysis of non-randomized studies to demonstrate that the biomarkers are “prognostic” with respect to treatment and control patients, respectively. In the control arm, g^+^ patients can be regarded as low-risk and g^−^ patients as high-risk subgroups. When both Comparisons 4 and 5 are of primary interest, the test strategy is to conduct Comparison 4 at the significance level of α in the first step; Comparison 5 is conducted at the significance level of α only if Comparison 4 is significant. Alternatively, type I error allocation of α_1_ and α_2_ can be used for the two comparisons. In addition, the mean or median estimates are useful to evaluate subgroup effect and treatment effect between g^+^ and g^−^ subgroups. Note that Comparison 5 is commonly used in assessment of prognostic biomarkers [30–32].

Simulation experiments were conducted to evaluate the proposed classification procedure and compare it to the ASD classifier. Binary responses are particularly useful for determination of classifier performance since predictive performance is well-measured by sensitivity, specificity, positive and negative predictive values, and accuracy. The evaluation focused on two aspects: 1) the statistical diagnostic test values of the classifiers, and 2) the power of the subgroup tests. The levels of significance were 2 % (one-sided) for the overall test and 3 % for the subgroup test [8]. Evaluation of the ASD classifier requires preselecting a list of plausible sets of tuning parameters to be used to search for “optimal” tuning parameters. We have reported the best performances for two parameters, R and G, denoted as ASD (ln(R),G).

The number of patients in each group was 200. For simplicity, the response probabilities for the g^+^ patients in the control group and for the g^−^ patients in the treatment group were assumed to be equal (u_10_ = u_01_). Eight scenarios were considered based on the 5 parameters: (π, u_00_, u_01_ = u_10_, u_11_, number of significant biomarker variables): A = (0.1, 0.2, 0.2, 0.6, 10), B = (0.1, 0.2, 0.2, 0.6, 15), C = (0.1, 0.2, 0.2, 0.6, 20), D = (0.1, 0.1, 0.2, 0.6, 10), E = (0.1, 0.2, 0.2, 0.8, 10), F = (0.1, 0.2, 0.4, 0.8, 10), G = (0.3, 0.4, 0.4, 0.8, 10), and H = (0.3, 0.4, 0.4, 0.8, 15). Two major themes were simulated, including equal response probabilities between g^+^ and g^−^ subgroups in the control (u_00_ = u_10_ = u_01_), and different response probabilities between them (u_00_ ≠ u_10_ = u_01_). The g^+^ patients in the treatment arm were generated from a Bernoulli random variable with probability u_11_, and the g^−^ patients in the control group were generated with probability u_00_. Other patients were generated with probability (u_01_ = u_10_). The expression variable was generated from a normal distribution with the mean of each probe generated using the formula: e^x^/(1 + e^x^) = u. The non-interacting genes were generated using the criteria: no mean difference between g^+^ and g^−^ patients in both control and treatment groups. The standard deviation was set at 0.3 for all variables. The total number of probes was 5,000 with three different numbers of predictive biomarkers (10, 15, and 20). The level of significance for the interaction test (Eq. 1) was set at 0.001. The DLDA algorithm was used to identify the g^+^ subgroup using the 10-fold cross validated ASD. For the ASD classifiers, we considered ln (R) = 1, 2 and G = 1, 2, denoted as (ln (R),G). These four cases represented the best performances for the two parameters R and G. This simulation was repeated 1,000 times.

All datasets analyzed in this study were published studies [32, 33] (Project id 182 in the ArrayExpress website [32] and GSE14814 in the Gene Expression Omnibus [33]).

## Results

### Simulation study

The DLDA algorithm showed the best predictive accuracy in all scenarios with reasonably good sensitivity and specificity, as well as positive and negative predictive values (Table 1). The one exception was a slightly lower accuracy value of 0.897 for DLDA than the value of 0.901 for ASD (2,2) in the scenario F = (0.1, 0.2, 0.4, 0.8, 10). For the ASD classifiers, small R or G is less restrictive in the selection of g^+^ patients resulting in high sensitivity and low specificity; conversely, large R and G will have low sensitivity and high specificity. Furthermore, the parameters R and G show that predictive accuracy varies for different scenarios; ASD (1,2) gives the highest accuracy for scenarios G and H, while ASD (2,2) gives the highest accuracy for scenarios A-F. This suggests that the selection of R and G parameters may depend on the prevalence proportion of the subgroup g^+^ (π). However, the proportion is usually unknown in practice, making it difficult to determine the optimal R and G parameters.

**Table 1:** Predictive performance of the five classifiers for eight scenarios (A-H). Each value is the average of 1,000 trials

Scenario	A	B	C	D	E	F	G	H
Predictive biomarkers	10	15	20	10	10	10	10	15
Significances	7.025	9.27	11.761	7.432	12.426	5.274	12.313	17.039
True positives	4.147	6.443	8.865	4.487	9.495	2.202	9.267	14.002
DLDA Sensitivity	0.598	0.651	0.694	0.626	0.979	0.507	0.985	0.99
Specificity	0.991	0.993	0.993	0.995	0.999	0.94	0.983	0.99
PPV	0.745	0.788	0.809	0.791	0.993	0.475	0.97	0.981
NPV	0.958	0.963	0.968	0.96	0.998	0.946	0.992	0.995
Accuracy	0.952	0.959	0.964	0.958	0.997	0.897	0.984	0.99
ASD (1,1) Sensitivity	0.784	0.808	0.841	0.943	0.991	0.953	0.989	0.991
Specificity	0.691	0.689	0.686	0.142	0.644	0.083	0.551	0.533
PPV	0.253	0.256	0.268	0.112	0.283	0.104	0.51	0.5
NPV	0.967	0.97	0.976	0.824	0.999	0.849	0.992	0.994
Accuracy	0.7	0.7	0.702	0.222	0.679	0.169	0.682	0.671
ASD (1,2) Sensitivity	0.635	0.683	0.723	0.831	0.982	0.807	0.977	0.982
Specificity	0.931	0.927	0.923	0.357	0.899	0.284	0.853	0.838
PPV	0.537	0.56	0.579	0.148	0.647	0.117	0.774	0.762
NPV	0.959	0.964	0.97	0.903	0.998	0.904	0.991	0.993
Accuracy	0.902	0.903	0.903	0.404	0.907	0.336	0.89	0.882
ASD (2,1) Sensitivity	0.464	0.533	0.573	0.766	0.953	0.654	0.581	0.649
Specificity	0.982	0.982	0.982	0.767	0.976	0.754	0.973	0.972
PPV	0.623	0.656	0.685	0.32	0.839	0.27	0.846	0.868
NPV	0.944	0.951	0.956	0.968	0.995	0.952	0.86	0.882
Accuracy	0.93	0.937	0.941	0.767	0.974	0.744	0.856	0.875
ASD (2,2) Sensitivity	0.331	0.408	0.453	0.64	0.926	0.445	0.424	0.511
Specificity	1	1	1	0.95	0.999	0.951	0.999	0.999
PPV	0.673	0.719	0.761	0.635	0.987	0.509	0.864	0.904
NPV	0.932	0.939	0.945	0.96	0.993	0.941	0.816	0.843
Accuracy	0.933	0.941	0.945	0.919	0.992	0.901	0.827	0.853

*PPV* positive prediction value, *NPV* negative prediction value

In scenario F, poorer prediction performances of all algorithms were observed due to the higher probabilities set for u_10_ = u_01_ in scenario 6. Higher values of u_10_ and u_01_ represent more noise in the response data. Similarly, dramatic changes in the accuracy values of ASD (1,1), ASD (1,2), and ASD (2,1) were observed in scenarios A and D, but the DLDA algorithm and ASD (2,2) showed similar or slightly lower accuracy values. These results suggest that the DLDA algorithm is less sensitive to changes at the population level, and that the ASD approach requires good selection of R and G parameters. It is not surprising that the DLDA algorithm showed higher accuracy values in scenarios A-C and G-H as the number of significant probes increased. However, this phenomenon was not observed in ASD (1,1) and ASD (1,2) in scenarios A-C and G-H. The reason may be that the ASD approach uses the voting method to classify patients; a higher number of identified probes cannot provide more information if the parameter G is not appropriate. In general, the DLDA algorithm shows high predictive accuracy in all scenarios, even though there are approximately 3 non-predictive probes in the model on average (data not shown). In scenario F, the number of predictive probes identified is less than 5 from Eq. 1, resulting in poor sensitivity. Notably, ASD (1,1) is the least restrictive classifier and has sensitivity equal to 0.953, the highest value, although specificity is only 0.083 in scenario F.

Empirical power of the overall and subgroup tests from five classifiers is summarized in Table 2. The overall power calculation was based on the original ASD paper published in 2005 [11] by summing the power of the overall test (Comparison 1 ≤ 0.02) and the power of the subgroup test (Comparison 2 ≤ 0.03 and Comparison 1 > 0.02). The power for DLDA to detect treatment effect in the g^+^ patients (Comparison 2) ranged from 0.3 to 0.5 in scenarios A-D and was greater than 0.9 in scenarios E and G-H when the overall test was not significant. Almost all 1,000 trials in scenario F were significant in the overall test (insignificant tests N = 3). In general, values for overall power demonstrated superior performance for the DLDA classifier compared to the ASD classifiers in all scenarios. Poor power in scenarios A-D resulted from low sensitivity in the selection of the g^+^ patients (Table 1); many g^+^ patients were misclassified into the g^−^ subgroup. For example, the ASD (1,1) classifier was the least restrictive in selecting g^+^ patients. Larger values of R or G will decrease the sensitivity and increase the specificity. In scenario E using ASD (1,1), sensitivity = 0.991 and specificity = 0.644, which implies that 0.356×180 = 64 g^−^ patients were classified as g^+^ patients in each group, where 13 would be responders and 51 would not. On average, the control group would be classified as having 17 responders and 67 non-responders and the treatment group would have 29 responders and 55 non-responders. The odds ratio for identifying treatment effect in the g^+^ patients was reduced to 2.08 from 16. Conversely, low sensitivity, as observed with ASD (2,2), would misclassify g^+^ patients as g^−^ patients. Either case can result in inadequate power to detect treatment effect in the g^+^ subgroup. In addition, PPV = 0.979 and NPV = 0.991 for the DLDA classifier in scenario E. Because PPV for the DLDA classifier was 0.979, only two g- patients would be misclassified as g + patients. Therefore, the DLDA algorithm indicated 20 responders in the control group and 79 responders in the treatment group. The odds ratio for identifying treatment effect in the g + patients was decreased to 15 from 16, which was substantially larger than 2.08 using the ASD (1.1) classifier. In summary, the DLDA classifier appeared to consistently perform better than the ASD classifiers.

**Table 2:** Power analysis for the overall test and subgroup tests of the five binary classifiers for eight scenarios (A-H). The overall power was calculated as the original reference [11], which is the sum of the number of overall test < 0.02 and the number of significance in comparison 2 of overall test > 0.02

Scenario	A	B	C	D	E	F	G	H
Overall Test < 0.02	64	70	69	924	159	997	463	487
Comparison 2_DLDA	26	35	33	483	153	503	455	481
ASD (1,1)	33	34	32	822	115	951	436	455
ASD (1,2)	20	25	25	682	116	859	451	476
ASD (2,1)	24	33	33	587	146	665	350	392
ASD (2,2)	23	33	31	488	153	411	286	352
Overall Test > 0.02	936	930	931	76	841	3	537	513
Comparison 2_DLDA	319	355	401	24	771	2	510	488
ASD (1,1)	130	131	115	11	298	0	388	361
ASD (1,2)	247	266	308	18	676	2	492	475
ASD (2,1)	216	260	280	20	709	2	226	237
ASD (2,2)	174	219	255	23	717	1	142	160
Overall power_DLDA	383	425	470	948	930	999	973	975
ASD (1,1)	194	201	184	935	457	997	851	848
ASD (1,2)	311	336	377	942	835	999	955	962
ASD (2,1)	280	330	349	944	868	999	689	724
ASD (2,2)	238	289	324	947	876	998	605	647

### Analysis of GSE14814 dataset

A total of 133 microarrays from GSE14814 [33] were downloaded from the GEO database. The data were from non-small-cell lung cancer patients; 62 patients received OBS alone and 71 patients received ACT. Because huge differences have been reported in distinct lung cancer subtypes, we divided the samples into two major subtypes, adenocarcinoma (AD, *N* = 71) and squamous cell carcinoma (SQ, *N* = 52). The logrank test between two treatment groups was performed for the cancer subtype. No significant survival differences were observed in either AD (*p* = 0.91) or SQ (*p* = 0.14) subtype. Since the AD patients showed almost no survival benefit from receiving the treatment, the 52 SQ patients were chosen for our analysis. There were 26 ACT patients and 26 OBS patients. For each gene, the Cox hazard regression model with an interaction term (Eq. 1) was tested. Because of small sample size, only the top 5 significant genes were selected as predictive biomarkers to develop the prediction model. The leave-one-out (LOO) cross validation was used in the analysis since some patients might have different subgroup assignments from different 10-fold partitions. The Cox regression model was fit to 51 training samples using all 5 significant genes to develop the prediction model. Subsequently, the pec library [34] in the R software was utilized to estimate the survival probability of at least 5 five years for the test patient. Probability of 0.5 was used as cut-off to select g^+^ and g^−^ patients. The procedure was repeated 52 times so that each patient was classified as either g^+^ or g^−^. For the ASD classifier, we considered the four best parameter settings: ln (R) = −1, −2, and G = 1, 2. Negative R values reflected reduced risk for the treatment group. For illustrative purpose, all five comparisons are reported in Table 3. Comparison 2 was not significant for all five classifiers. The number of patients and the median survival for each of the four groups are listed in the last four rows. Note that the median survival times for OBS and ACT were 5.34 and 6.69, respectively. The p-values from Comparison 4 are also shown. The performances of DLDA and ASD (−1,1) were very similar. Both analyses showed that the patients in the control arms had longer median survival time than the patients in the treatment arm for the g^−^patients identified.

**Table 3:** Subgroup identification and analysis of 52 squamous cell carcinoma patients using a leave-one-out cross validation for five classification methods. There were 26 patients in ACT and 26 patients in OBS

	DLDA	ASD (−1,1)	ASD (−1,2)	ASD (−2,1)	ASD (−2,2)
Comparison 1	0.138	0.138	0.138	0.138	0.138
Comparison 2	0.07	0.125	0.149	0.428	0.114
Comparison 3	0.039	0.083	0.847	0.125	0.008
Comparison 4	1.25E-08	0.001	0.689	0.267	0.143
Comparison 5	0.697	0.697	0.938	0.791	0.034
g+ (T)^a^	6.79 (24)	6.71 (25)	6.71 (23)	6.71 (17)	5.21 (6)
g+ (C)	5.68 (23)	5.68 (23)	5.44 (22)	3.00 (15)	6.59 (8)
g− (T)	1.21 (2)	1.32 (1)	6.54 (3)	6.54 (9)	6.79 (20)
g− (C)	3.17 (3)	3.17 (3)	4.43 (4)	5.68 (11)	3.09 (18)

Each method classified patients into 4 subgroups: g+ (T), g+ (C) g− (T) and g− (C)

^a^Median survival time (year) in the subgroup and the number of patients in the subgroup in parentheses

### Retrospective analysis of Shedden’s lung adenocarcinoma dataset

We analyzed a well-known lung AD dataset [32]. In this study, a favorable survival difference was observed in the control group (*p* = 1.14*10^−5^). For illustrative purposes, this dataset was analyzed as a binary outcome, based on two year survival time. Comparisons 4 and 5 were conducted to identify potential predictive biomarkers and potential predictive-prognostic biomarkers. Two hundred thirty-two (232) patients with clear “death” status were analyzed. Patients were dichotomized into two groups based on whether or not their survival was greater than 2 years. There were 80 patients in the control group and 152 patients in the treatment group. Similar to the analysis performed in the GSE14814 dataset, a LOO cross-validation analysis was performed.

The DLDA and four ASD classifiers, ASD (−0.5,1), ASD (−0.5,2), ASD (−1,1), and ASD (−1,2), were developed to identify g + patients. All five comparisons are reported in Table 4. The DLDA algorithm showed a significant survival difference between g^+^ and g^−^ patients in the treatment group (Comparison 4) and no difference in the control group (Comparison 5). Among the four ASD classifiers, only ASD (−0.5,1) showed a survival difference in Comparison 4 close to borderline significance (*p* = 0.049). The survival difference between the g^+^ and g^−^ subgroups in DLDA was 1.04, while the difference in ASD (−0.5,1) was 0.97. ASD (−0.5,2) also showed an obvious survival difference between g^+^ and g^−^ patients in the treatment group (Comparison 4). However, the g^−^ subgroup for the control group contained no patients. For ASD (−1,1) and ASD (−1,2), no patients had an ln (R) smaller than −1.

**Table 4:** Retrospective analysis of 232 lung adenocarcinoma patients by five binary classifiers. There were 80 patients in the control and 152 patients in the treatment

	DLDA	ASD (−0.5,1)	ASD (−0.5,2)	ASD (−1,1)	ASD (−1,2)
Comparison 1	0.014	0.014	0.014	0.014	0.014
Comparison 4	0.018	0.049	0.014	NA	NA
Comparison 5	0.86	0.024	NA	NA	NA
Comparison 2	0.578	0.739	NA	NA	NA
Comparison 3	0.432	0.061	0.024	0.014	0.014
g+ (T)	3.40 (55)^a^	2.95 (118)	2.80 (146)	1.84 (152)	1.84 (152)
g+ (C)	2.36 (97)	1.98 (34)	1.16 (6)	NA (0)	NA (0)
g− (T)	3.82 (73)	3.89 (75)	3.46 (80)	3.46 (80)	3.46 (80)
g− (C)	3.35 (7)	2.02 (5)	NA (0)	NA (0)	NA (0)

^a^Median survival time (year) in the subgroup and the number of patients in the subgroup in parentheses

## Discussion

Both the ASD and the proposed classifier used the same predictor biomarker set to develop a classifier. The ASD classifier consisted of a set of base-classifiers, and each base-classifier used only a single biomarker to select g^+^ patients. The base-classifiers used the same cutoff for all biomarkers which may not be an optimal strategy. Furthermore, the classifier may be sensitive to a “super” predictor which may classify all patients as g^+^. The proposed classifiers combined all predictors by finding the best weights to develop a prediction model and determine a cutoff to classify g^+^ and g^−^ patients. The proposed classifiers are developed by learning the relationship between the predictors and subgroup memberships. When there are several potential predictors, using all predictors to develop a classifier is the standard and effective approach. The proposed classifier is expected to have better performance than the ASD classifier (Tables 1 and 2).

Eight simulation scenarios with different parameters were considered in this study. It is not surprising to observe different accuracy and power values under distinct settings. One limitation in the simulation scenarios is that the g^+^ patients were assumed to be homogeneous. That is, only one g^+^ subgroup was simulated, which may not be applicable in the real world since patients are very heterogeneous. Further research and simulation study may be conducted to evaluate the predictive performances with more than one g^+^ subgroup.

Subgroup analysis in clinical trials commonly refers to Comparisons 1 and 2, where there are beneficial effects in all patients and in a subgroup [35, 36], given that the subgroups have been well-defined and correctly classified. The analysis strategy starts with an interaction test for differential treatment effect between subgroups [37], and subsequently performs subgroup identification and analysis. It is generally expected that the prevalence for the g^+^ subgroup is less than 50 %. Therefore, a significant outcome of Comparison 2 often implies a significant outcome for Comparison 3, provided that the classifier has a high specificity (Table 1). Comparison 3 provides additional information regarding the labeling effect in the g^−^ subgroup when Comparison 1 is significant.

Prospectively planned biomarker adaptive design provides a useful tool to assess a new treatment effect in all patients and in biomarker defined targeted patients. It assumes two underlying subgroups in the patient populations with greater treatment efficacy in the biomarker targeted subgroup. The designs combine subgroup identification and subgroup analysis in one study. Accurate identification of subgroups is critical to the success of the study. For continuous survival data, subgroup identification involves estimation of predictive scores and selection of a threshold for subgroup classification. Recently, tree-based methods of directly finding treatment–covariate interactions have been proposed [38–45]. Tree-based methods identify biomarkers while classifying patients into subgroups; many such methods were exploratory analysis to identify predictive biomarkers that showed treatment effects in subgroups; on the other hand, the ASD framework was proposed for confirmatory analysis to make inferences about a target subgroup. Various methods have been proposed to estimate predictive scores and select a threshold. The determination of optimal weights to estimate predictive scores and the threshold cut-point for subgroup classification remains a significant challenge. Further research in prediction models for classification of survival response data is warranted [11].

Another major challenge is the allocation of α_1_ and α_2_ that significantly impact the interpretation and conclusion of the trial. An optimal allocation depends on the characteristics of the two subpopulations and on the treatment effect sizes for the two subpopulations. Research to develop testing strategies for the three populations (all population, targeted population, and non-targeted population) and to determine sample size will help in the decision strategy for subgroup-specific treatment effects in the context of biomarker adaptive design.

## Conclusion

In this study, we presented new procedures to classify patients into different subgroups to detect their treatment effects based on gene expression values. A simulation study and two real datasets were analyzed to demonstrate superior performance and accuracy when being compared with a published method. In summary, the results showed that the proposed design is an effective approach to identify subgroups of patients and to determine their ability to benefit from a treatment.

## Abbreviations

AD: adenocarcinoma
ASD: adaptive signature design
IVD: in vitro
LDA: linear discriminant analysis
LOO: leave-one-out
NPV: negative prediction value
PPV: positive prediction value
QDA: quadratic discriminant analysis
SQ: squamous cell carcinoma
